# Microstructure and Mechanical Characteristics of Ti-Ta Alloys before and after NaOH Treatment and Their Behavior in Simulated Body Fluid

**DOI:** 10.3390/ma16051943

**Published:** 2023-02-26

**Authors:** Iosif Hulka, Julia Claudia Mirza-Rosca, Dragos Buzdugan, Adriana Saceleanu

**Affiliations:** 1 Department of Mechanical Engineering, Las Palmas de Gran Canaria University, 35017 Las Palmas de Gran Canaria, Spain; 2 Research Institute for Renewable Energy, Politehnica University Timisoara, 138 Gavril Musicescu Street, 300774 Timisoara, Romania; 3 Department of Materials and Manufacturing Engineering, Politehnica University Timișoara, Piața Victoriei, No. 2, 300006 Timișoara, Romania; 4 Medicine Faculty, “Lucian Blaga” University of Sibiu, 550024 Sibiu, Romania

**Keywords:** biomaterials, mechanical properties, alkali treatment, titanium-tantalum alloys

## Abstract

In the present study, the microstructure and mechanical properties of Ti-xTa (x = 5%, 15%, and 25% wt. Ta) alloys produced by using an induced furnace by the cold crucible levitation fusion technique were investigated and compared. The microstructure was examined by scanning electron microscopy and X-ray diffraction. The alloys present a microstructure characterized by the α′ lamellar structure in a matrix of the transformed β phase. From the bulk materials, the samples for the tensile tests were prepared and based on the results and the elastic modulus was calculated by deducting the lowest values for the Ti-25Ta alloy. Moreover, a surface alkali treatment functionalization was performed using 10 M NaOH. The microstructure of the new developed films on the surface of the Ti-xTa alloys was investigated by scanning electron microscopy and the chemical analysis revealed the formation of sodium titanate and sodium tantanate along with titanium and tantalum oxides. Using low loads, the Vickers hardness test revealed increased hardness values for the alkali-treated samples. After exposure to simulated body fluid, phosphorus and calcium were identified on the surface of the new developed film, indicating the development of apatite. The corrosion resistance was evaluated by open cell potential measurements in simulated body fluid before and after NaOH treatment. The tests were performed at 22 °C as well as at 40 °C, simulating fever. The results show that the Ta content has a detrimental effect on the investigated alloys’ microstructure, hardness, elastic modulus, and corrosion behavior.

## 1. Introduction

In the field of biocompatible materials, commercially pure (CP) Ti is extensively used for the manufacturing of implants. Nevertheless, it has some drawbacks such as low wear caused by joint movement, low degree of deformability, etc. [1]. In order to overcome these issues, Ti alloys have become a popular choice for implants since they have high mechanical reliability [2], improved properties in terms of low specific weight, and increased corrosion resistance [3]. The corrosion resistance is vital for biomaterials since the ions released during corrosion can reduce the implant biocompatibility and can produce an unwanted side reaction in peri-implant tissues, leading to inflammation, lowering the mechanical properties, and even implant failure can occur [4]. The corrosion behavior of biomaterials is strongly influenced by the passive oxide layer developed on its surface when it is in contact with the physiological environment. Thus, the implants should be composed of non-toxic elements with low toxicity and without causing allergic problems. Moreover, the implants require a reduced Young’s modulus, close to that of the bone which is in the range of 10–30 GPa to prevent bone resorption which leads to bone remodeling [5]. If the Young’s modulus of the implant differs greatly from that of the bone, it may result in a stress-shielding effect which might require revision surgery [6]. Among the non-toxic and non-allergic alloying elements used in Ti alloys that meet the mentioned conditions, the most common are Nb, Zr, Mo, and Ta [7,8]. Alloyed with Ti, Ta is considered one of the greatest biomaterials due to its excellent corrosion resistance. Its high resistance to corrosion, even when exposed to hot and concentrated mineral-reducing acids, is attributed to the development of a protective Ta_2_O_5_ film [9]. The bond between the surface of the biomaterial and the surrounding tissues is conditioned by the tissue fluids and plasma. This leads to the development of a macromolecules layer, water and proteins which is responsible for the behavior of cells when they encounter the biomaterial. In addition, an increased surface roughness leads to a strong interaction between the implant and bone by increasing the contact area.

In order to increase the cohesion and strength with the bone, several methods have been employed such as the deposition of hydroxyapatite (HA) by various methods. Dhinasekaran et al. used a pulse laser deposition technique to develop HA coatings on CP Ti [10] while Lin et al. deposited HA coatings on Ti plates using ultrasonic mechanical coating and armoring [11]. Other deposition methods for HA coatings are axial plasma spraying [12] and high velocity oxygen fuel spraying [13]. Moreover, sputtered HA thin films were successfully obtained by Oladijo et al. to enhance the biocompatibility of stainless-steel implants [14]. However, the unpredictable binding between hydroxyapatite coatings or films and the Ti-based alloys raised concerns about the long-term durability within the human body [15]. Therefore, an alternative method to enhance the quality of bonding between the bioactive film and the implant is to activate the implant surface by using NaOH solution.

To activate the implant surface by providing micro- and nano-roughness on Ti implants, chemical treatment in alkali solution can be employed as an alternative to the well-known sand blasting technique followed by acid etching [16]. Jalota et al. successfully developed carbonated apatitic calcium phosphate on a Ti6Al4V substrate from simulated body fluid by using 5 M NaOH solution at 60 °C for 24 h [17]. Wang et al. presented a bone-like apatite formation on the surface of Ti6Al4V treated with NaOH solution and they discovered that electrochemical impedance spectroscopy is a useful method to investigate the nucleation and growth of apatite in simulated body fluid [18]. Similar findings were reported by He et al. when Ti6Al4V was pretreated with 8 M NaOH solution at 60 °C for 48 h [19]. Thus, the chemical composition of the surface of the implant may lead to bone binding and affinity by developing hydroxyapatite precipitates from body fluid [20].

The compositions of Ti-xTa alloys in this work were selected in order to further study the microstructure and mechanical properties of these alloys obtained by the cold crucible levitation fusion technique which are strong candidates as biomaterials. Thus, the aim of the present work is to investigate the influence of the functionalization treatment on the microstructure and mechanical properties of Ti-xTa alloys. The surface functionalization by 10 M NaOH alkali treatment was performed in order to study the osteogenic potential of the alloys. So far, studies were conducted generally on Ti6Al4V. The influence of the functionalized layer on the hardness behavior was studied as well. After the surface treatment functionalization, the samples were immersed in Ringer’s solution in order to improve the osseoinductive activity of the samples. Moreover, the behavior was evaluated in Ringer’s solution before and after the surfaces were treated using NaOH. The tests were performed at 22 °C as well as at 40 °C, simulating fever conditions. Even though the literature includes studies regarding the me-chemical and microstructural characterization and corrosion behavior of Ti-xTa (x = 5, 15, 25% wt. Ta) alloys, as far as we are aware, there are no studies regarding the elastic modulus determined using tensile tests for very small sample, Ti-xTa surface functionalization by using 10 M NaOH in order to increase the biological activity in the alloys nor in vitro studies after surface treatment simulating fever conditions which is an accelerated process due to the increased temperature.

## 2. Materials and Methods

### 2.1. Material Preparation

The binary Ti-Ta alloys with different Ta contents (5%Ta, 15%Ta and 25%Ta) were prepared using an induced furnace by the cold crucible levitation fusion technique. Argon was used as shielding gas in order to avoid oxidation. The alloys were manufactured under ingot shape with a diameter of 20 mm and a length of 30 mm. Homogenization was performed to avoid segregation by performing heat treatment in a tubular furnace at 1000 °C, with 5 °C/min heating rate followed by cooling in air.

### 2.2. Microscopic Observations

The samples were embedded in carbon-based resin and afterward polished with SiC abrasive papers in the range of 280–2500 P and then with alumina paste until a mirror-like surface was obtained. The samples were cleaned with alcohol in an ultrasonic bath followed by etching using Kroll’s reagent. The metallographic samples were investigated by scanning electron microscopy (SEM, Zeiss Sigma 300 VP, Carl Zeiss, Jena, Germany). The SEM was operated in high vacuum mode at a cathode voltage of 20 kV using the secondary electron detector (SE). Energy dispersive X-ray (EDS) was employed as well in order to determine the chemical composition of the alloys after homogenization.

### 2.3. Phase Structure

In order to study the phase structure of the investigated alloys, X-ray diffraction was performed on an Empyrean diffractometer (Malvern-Panalytical, Malvern, UK). The measurements were performed on polished samples at 22 °C using Cu Kα radiation at an angle of 2θ in the range of 30–65°. The step size employed was 0.04° at a power of 45 kV.

### 2.4. Surface Treatment with 10 M NaOH

In order to improve the osteoinduction, a superficial treatment was employed by immersing the investigated samples in 100 mL of NaOH 10 M at 60 °C for 24 h and its effect on the micro-hardness and corrosion behavior was studied. The functionalized surfaces were investigated by SEM (Quanta FEG 250, FEI, Hillsboro, OR, USA) using backscattered electron detector (BSD) equipped with an EDS spectrometer employed to evaluate the chemical composition of the treated surfaces.

### 2.5. Micro-Hardness Measurements

The micro-hardness of the alloys was measured using a Buehler Micromet VD 5124 (Buehler, Lake Bluff, IL, USA) hardness tester with a Vickers indenter. A low load of 49 mN was employed for the study in order to compare the hardness of the initial samples and to measure the hardness of the layer developed on the surface of the samples after the NaOH treatment. A 15 s holding time was used and 10 measurements were performed on each sample. 

### 2.6. Elastic Modulus Obtained from Tensile Test

**Figure 1:**
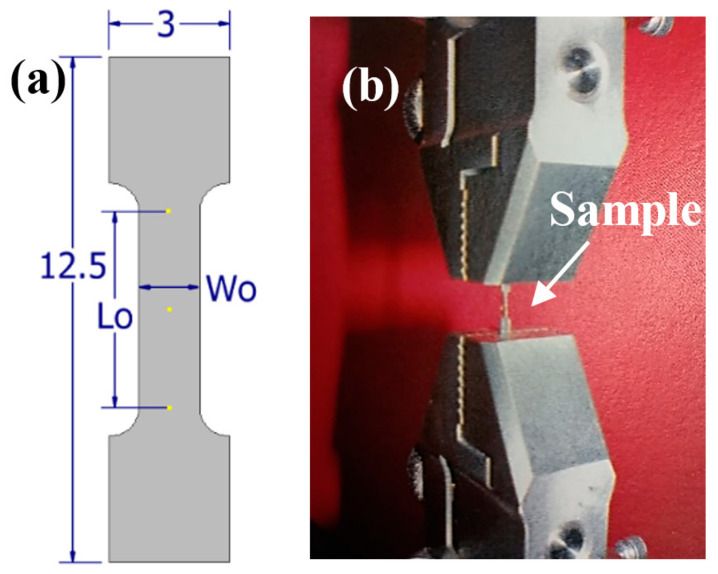
Schematic of tensile test specimens (**a**) and dog-bone-shaped sample fixed in the tensile testing machine (**b**).

The tensile tests were performed on an ElectroForce 3100 (Bose, Eden Prairie, MN, USA) tensile testing machine at 22 °C. The samples were prepared with the size of gauge length (L_0_), width (W_0_), and thickness (T_0_) presented in Table 1. The samples were cut from ingots using an IsoMet 4000 linear precision saw (Buehler, Lake Bluff, IL, USA) followed by fine polishing until the desired shape was obtained (Figure 1a). The shoulders of the samples were firmly and carefully gripped by the tensile tester (Figure 1b) in order to avoid damage of the fine specimens. Three samples were prepared for each alloy to ensure repeatability of the measured results.

The samples with the dimensions presented in Table 1, including the cross-sectional area (S_0_), were subjected to tensile tests using a maximum force of 22 N using a constant speed of 0.8 N/s.

Based on the tensile test results, the elastic modulus was calculated using the following equation:

(1) $$E_{t}=\frac{F/S_{0}}{\Delta L/L_{0}}\;\;\;$$

where *F* is the applied force, *S*_0_ is the cross-sectional area, *L*_0_ is the gauge length of the test specimen, *W*_0_ represents the width of the samples, and Δ*L* is the change in the gauge length.

### 2.7. Corrosion Behavior

The corrosion behavior of the tested specimens was determined by open circuit potential (OCP) measurements carried out by using a BioLogic SP-150 Potentiostat (BioLogic, Seyssinet, France). The testing technique was performed on samples with and without 10 M NaOH treatment, in both cases at 22 °C and at 40 °C, in order to study the feverish state of persons who might have an implant manufactured from a Ti-xTa alloy. The results were obtained in the form of graphs by using the Power Suit software representing the corrosion potential values (V) versus time (s). The tests were performed over a period of 24 h, by taking measurements at every 43.2 s.

## 3. Results and Discussions

### 3.1. Microstructure of Ti-xTa Alloys

**Figure 2:**
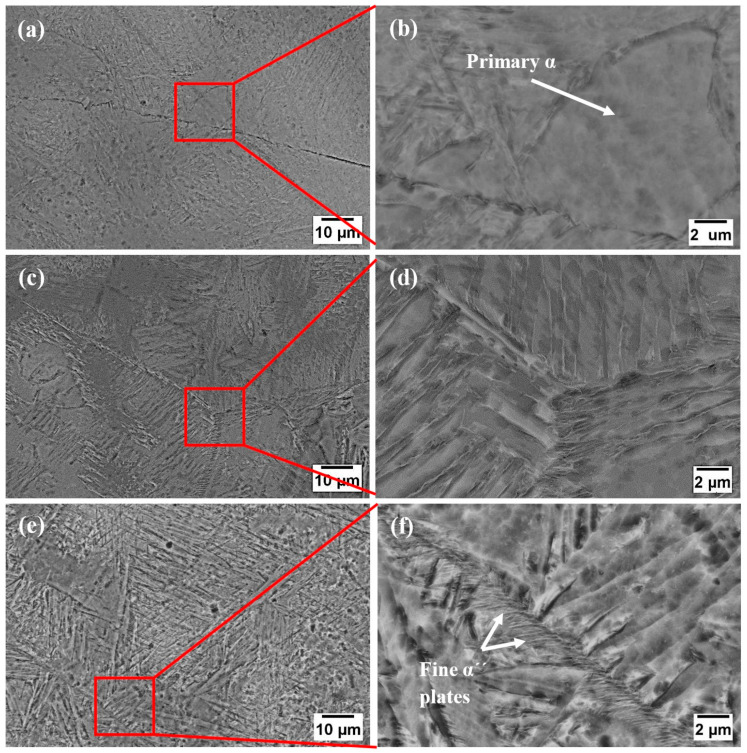
Microstructure of Ti-xTa alloys on SEM—(**a**,**b**) Ti-5%Ta; (**c**,**d**) Ti-15%Ta; (**e**,**f**) Ti-25%Ta.

The SEM micrographs representing the different microstructures of the studied alloys are presented in Figure 2. It can be seen that the microstructure is very sensitive to the Ta addition and evolved with the increase in the Ta content. Ta is a β phase stabilizer in Ti-based alloys and their microstructure consists generally of α and β phases alongside α′ and α″ phases which are non-equilibrium martensitic phases [21]. The Ti-5Ta sample presents a microstructure characterized by the α′ lamellar structure in a matrix of transformed β containing equiaxed primary α. By increasing the Ta content to 15%, the microstructure presents a lamellar α′ structure in the β phase. From the high magnification micrographs presented in Figure 2 labeled (f), it can be observed that the lamellar α′ and acicular α″ phases occur when the Ta content is added in a higher amount (25% Ta). The α″ phase precipitated preferentially at the grain boundaries, reported as well by Yumak et al. [22]. The supersaturated α′ and α″ solid solutions formed due to quenching from β when the samples were taken from the oven and air-cooled.

**Figure 3:**
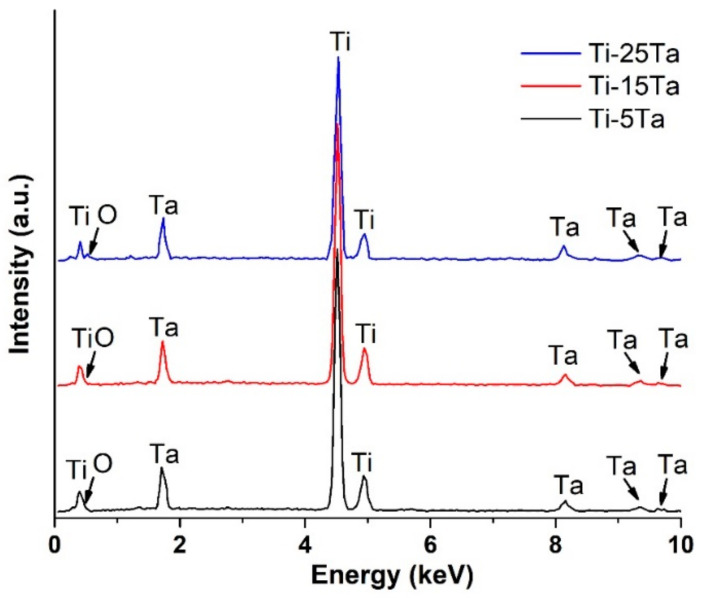
EDS spectra of the investigated alloys.

The EDS analysis was performed on the investigated alloys and the associated spectra are presented in Figure 3. From the investigations, it can be seen that there are no impurities in the alloys; therefore, only Ti and Ta were identified along with some traces of oxygen. Moreover, the spectra show that the alloys are homogenous and free of segregations. The presence of oxygen is due to the high affinity of this element to Ti and Ta which developed the oxide layer when exposed to the environment.

**Figure 4:**
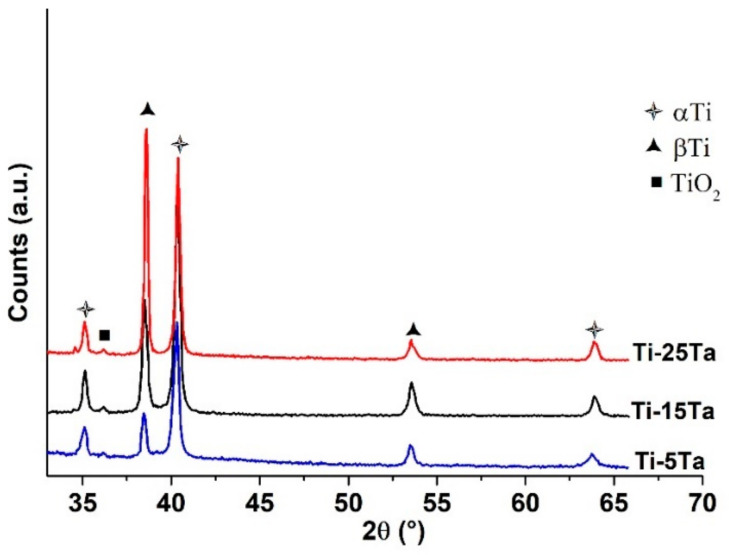
XRD spectra of the investigated samples: blue—Ti-5%Ta, black—Ti-15%Ta and red—Ti-25%Ta.

The XRD patterns of the investigated samples are presented and compared in Figure 4. For all three samples, peaks of the α and β phases were detected. A similar finding was reported in our previous studies [23]. However, since the hexagonal α′ and orthorhombic α″ martensitic phases are very close in the XRD pattern, it is very difficult to distinguish them by using phase analysis. Thus, in the XRD pattern, only the α phase was used as annotation. In analyzing the patterns, one can notice that the α phase is higher in the alloy containing lower amounts of Ta compared to the β phase, indicating that the α phase predominates the microstructure which is in accordance with the microstructural characterization. In the samples containing 15% and 25% Ta, an increase in the β phase diffraction peak was observed and this is attributed to the fact that Ta, which is a β stabilizing element, leads to an increased β volume fraction within the alloys. A minor peak was detected at the position 36θ which is attributed to the TiO_2_ phase which forms as a protective and self-adherent film on the surface of Ti [9]. XRD failed to detect other phases which might be present in the microstructure such as the ω phase due to their small presence.

### 3.2. Elastic Modulus Calculated from Tensile Test Results

**Figure 5:**
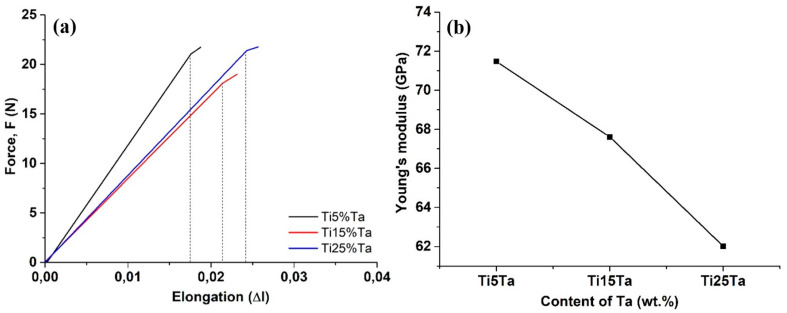
Force–elongation curves of the tested samples (**a**) and variation in elastic modulus with Ta content (**b**).

The elastic modulus was calculated based on the tensile test results. Figure 5 labeled (a) presents the force–elongation curves obtained for the Ti-x%Ta samples when applying a load of 22 N. The strength variation in the Ti-Ta alloys is due to the microstructural change caused by the Ta content. The finer microstructure of the α phase of the Ti-25%Ta contributed to the increased strength of this composition. Additionally, the degree of saturation of α increases with the Ta content, leading to an improved solid solution strengthening within the alloy [24]. The data used for the calculus of the elastic modulus were determined based on the linear slope presented in the force–elongation curves which end at the yield point. After this point, one can notice that a permanent plastic deformation occurs in the material under the applied force. The linear elastic behavior varied the function of the Ta content and it had a direct effect on the elongation of the samples. After the elastic behavior, a transient area from an elastic to quasi-plastic–elastic area can be observed. Similar observations were reported by Suttner et al. when determining the elastic modulus from uniaxial tensile tests of sheet metals [25]. Using Hook’s equation presented at paragraph 2.6, the elastic modulus was calculated, and the results are presented in Figure 5b. It can be observed that the elastic modulus decreases linearly with the increase in the Ta content. Thus, the lowest elastic modulus among the investigated samples was calculated for Ti-25Ta and, as can be observed, a value of 62 GPa was obtained. Similar findings were reported by Zhou et al. when studying the mechanical properties of Ti-25Ta in comparison with other Ti-Ta alloys. They reported that the Ti-25Ta had the lowest elastic modulus among the studied compositions with a value of 64 GPa [26]. However, the calculus of the elastic modulus from the tensile tests presents variations and scatter in comparison with the dynamic measurements [27], which might be the reason for the different values obtained compared to the literature. Therefore, the calculus of the elastic modulus from the tensile tests performed on very small samples at forces up to 20 N might be a promising method to provide information about the mechanical properties of biomaterials.

### 3.3. Microstructure of the Functionalized Surfaces after NaOH Treatment

**Figure 6:**
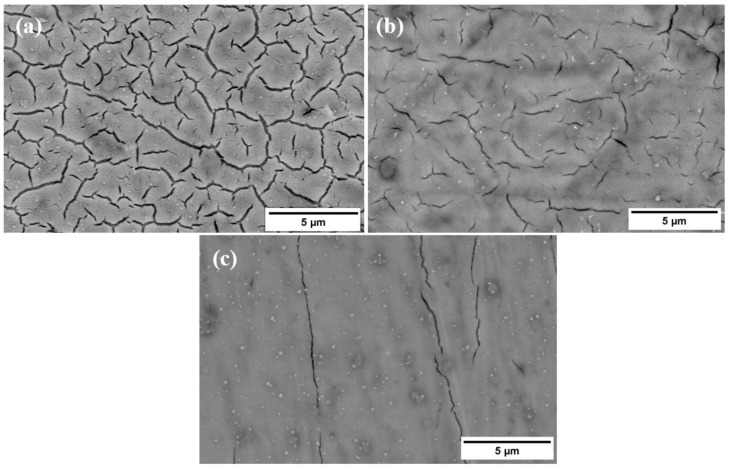
Microstructure of Ti-xTa substrates treated with 10 M NaOH at 20 k magnification—(**a**) Ti-5%Ta; (**b**) Ti-15%Ta; (**c**) Ti-25%Ta.

The surface morphology after treatment with NaOH solution was studied by SEM. The treated surfaces, presented in Figure 6, exhibit different morphologies. Since the alloys were treated in the same way using the same concentration of NaOH and same exposure time, it is believed that the various structures are directly influenced by the Ta content within the substrate. Thus, with an increase in the Ta content, a smoother film surface is obtained, as presented in the SEM images collected at 20 k magnification.

**Figure 7:**
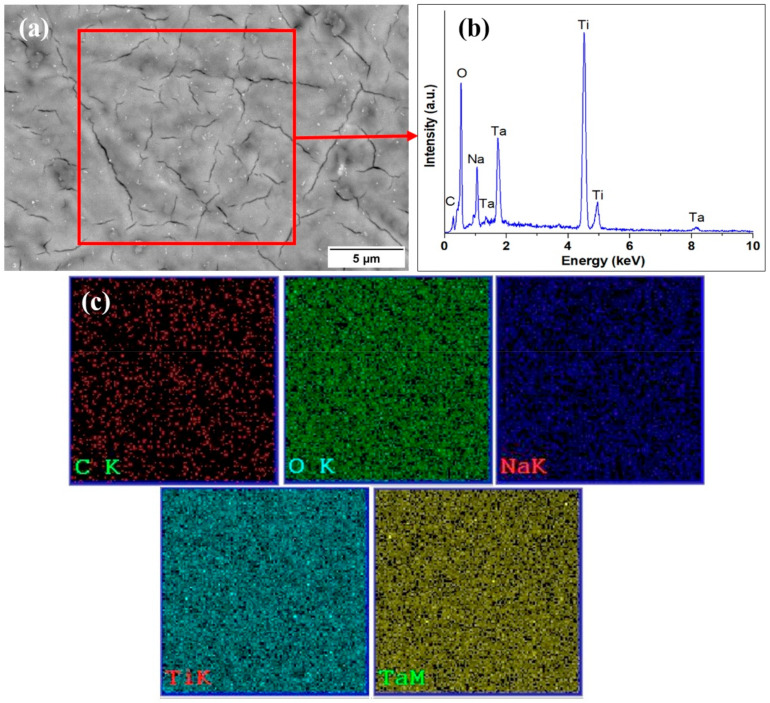
Microstructure of Ti-15Ta substrate treated with 10 M NaOH with afferent chemical composition—(**a**) Ti-15%Ta; (**b**) EDS on selected area; (**c**) elemental mapping.

After soaking in NaOH solution, a new film was developed on the surface of the samples composed mainly of sodium titanate and sodium tantanate along with titanium and tantalum oxides, as was revealed by the EDS analysis. In a similar study, Luo et al. used 5 M NaOH to functionalize a Ti6Al4V substrate and a rich sodium titanate film was obtained. Since the chemical composition was similar for the investigated samples, only the results for Ti-15Ta are presented in Figure 7.

### 3.4. Composition of the Functionalized Surfaces after Exposure to Simulated Body Fluid

**Figure 8:**
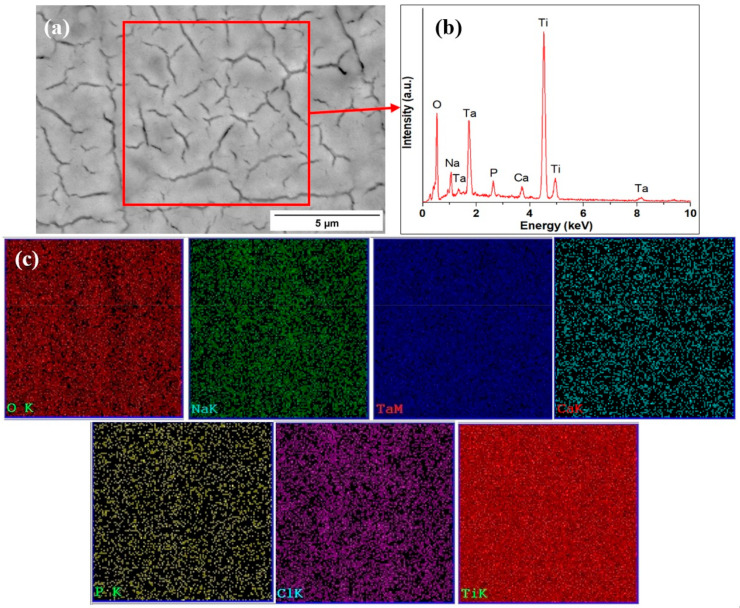
Microstructure of Ti-15Ta substrate treated with 10 M NaOH after exposure to simulated body fluid with afferent chemical composition—(**a**) Ti-15%Ta; (**b**) EDS on selected area; (**c**) elemental mapping.

After exposure to Ringer’s solution, the microstructure of the treated alloys did not change significantly, as can be observed in Figure 8. Because of this, only the microstructure of the Ti-15Ta sample was presented. However, it can be observed that the chemical composition has changed, and Ca and P were detected on the surface of the treated samples, dispersed homogenously, according to the EDS mapping.

### 3.5. Hardness Evaluation

Comparing the results presented in Table 2, it can be observed that the hardness values increase from 155.34 (Ti-5Ta) to 219.69 (Ti-25Ta). The higher hardness value for the Ti-25Ta composition is attributed to an improved structural hardening caused by an ideal distribution of the α phase within the β phase [28]. The hardness values after the NaOH treatment increased considerably, as presented in Table 2. During the NaOH treatment, the stable TiO_2_ reacts with the solution and forms a sodium titanate film while the Ta_2_O_5_ forms a sodium tantanate amorphous film. However, not all the amount of TiO_2_ and Ta_2_O_5_ reacts during the process. Higher hardness values for the treated surfaces might be attributed to the formation of sodium titanate and sodium tantanate combined with Ti and Ta oxides after the NaOH treatment. It was reported that oxides developed on Ti alloys have higher hardness values compared to the non-treated surface [29]. Nevertheless, the results are for low testing loads which were employed during the study in order to avoid damaging the new layer developed after the NaOH treatment.

## 4. Corrosion Tests

Metals immersed in an electrolytic medium generate an electric potential (open circuit potential, OCP) that varies as a function of immersion time. This potential stabilizes and remains constant after immersion for a certain time. The variation in the potential over time occurs due to processes occurring on the surface of the electrode when in contact with an electrolyte which produces changes in the nature of the electrode surface. The shift in the corrosion potential in the positive direction (anodic direction) can be associated either with an acceleration of the reduction reaction (cathodic reaction) or with an inhibition of the oxidation reaction (anodic reaction). The inversion or displacement of the corrosion potential in the negative direction (cathodic direction) may correspond either to an acceleration of the oxidation reaction or to an inhibition of the reduction reaction. In general, it can be established that the evolution of the potential in the positive direction corresponds to the formation of a protective oxide layer on the surface, and when it moves toward negative values it can be associated with a reorganization of the surface layer of its internal configuration with an increasingly lower resistance to corrosion [30]. Alloys with the most negative potentials will generally tend to suffer more significant corrosion than other alloys (with positive potentials) which will suffer less corrosion attack. Thus, the open circuit potential is a useful method to evaluate the corrosion behavior of alloys.

**Figure 9:**
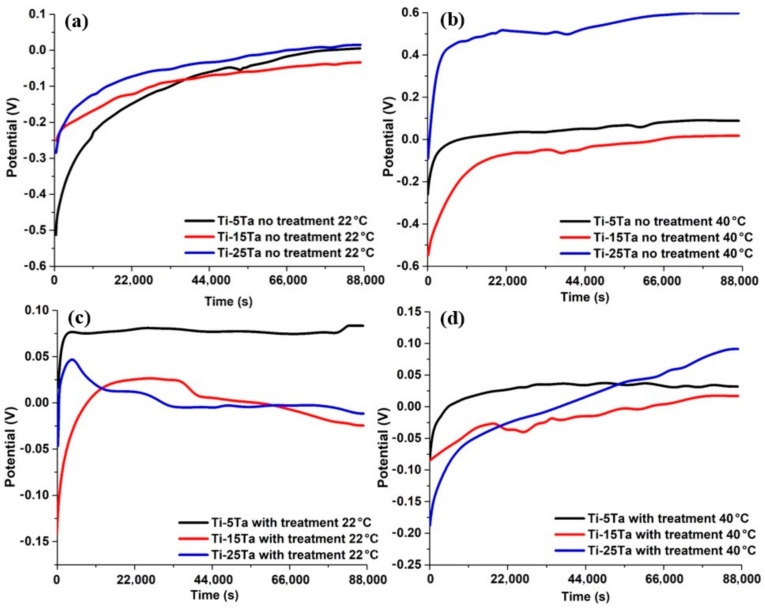
Open circuit potential curves for Ti-xTa alloys during 24 h immersion in the electrolyte in various conditions: (**a**) OCP results for samples without NaOH treatment at 22 °C; (**b**) OCP results for samples without NaOH treatment at 40 °C; (**c**) OCP results for samples with NaOH treatment at 22 °C; and (**d**) OCP results for samples with NaOH treatment at 40 °C.

As a follow-up, Figure 9 presents the OCP results for various conditions as follows: (a) OCP results for the samples without the NaOH treatment at 22 °C; (b) OCP results for the samples without the NaOH treatment at 40 °C; (c) OCP results for the samples with the NaOH treatment at 22 °C; and (d) OCP results for the samples with the NaOH treatment at 40 °C.

In Figure 9a, it can be observed that for all the samples, the open circuit potential increases, reaching almost the same stable value for Ti-5Ta and Ti-25Ta and a little lower for Ti-15Ta [23,31] after 24 h of immersion. At 40 °C (see Figure 9b), the differences between the stable values of the open circuit potential for the three alloys are more pronounced. The open circuit potential increasing with time is due to the following reactions which take place at the samples’ surface:

(2) $$\mathrm{Ti}+2H_{2}O={\mathrm{TiO}}_{2}+4H^{+}+4e^{-}$$

(3) $$2\mathrm{Ta}+5H_{2}O={\;\mathrm{Ta}}_{2}O_{5}+10H^{+}+10e^{-}\;$$

**Figure 10:**
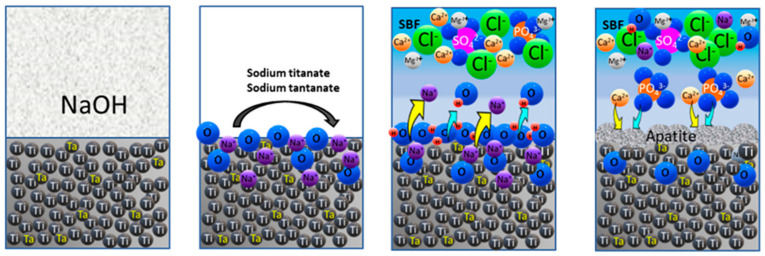
Schematics of apatite formation on Ti-xTa.

With the NaOH treatment and posterior heat treatment, at the surface of the samples, a film of sodium titanate and sodium tantanate is formed [32,33]. At 22 °C (see Figure 9c), a sharp increase in the potential can be observed for all the samples during the first hour of immersion; after this period, the OCP is mainly stable for Ti-5Ta but decreases significantly for Ti-15Ta and Ti-25Ta due to the interactions of sodium titanate and tantanate with the calcium and phosphate ions from the simulated body fluid. At 40 °C (see Figure 9d), these processes are taking place rapidly, resulting in apatite formation (the processes are represented in Figure 10).

## 5. Conclusions

In the present study, Ti-xTa (x = 5%, 15%, and 25% wt. Ta) alloys produced by using an induced furnace by the cold crucible levitation fusion technique were investigated. The samples were subjected to a 10 M NaOH treatment and the microstructure and mechanical properties were evaluated at 22 °C as well as at 40 °C, simulating fever conditions. Thus, by modifying the Ta content within the alloy, the microstructure can be changed from a microstructure characterized by the α′ lamellar structure in a matrix of transformed β containing equiaxed primary α for the Ti-5Ta alloy, to a microstructure with the lamellar α′ structure in the β phase, with fine α″ acicular structures precipitated preferentially at the grain boundaries for the Ti-25Ta alloy. EDS analysis shows only Ti and Ta within the microstructures along with some traces of oxygen, indicating that the alloys are homogenous and free of segregation. According to the XRD patterns, an increase in the Ta content to 15% and 25% leads to the growth in the β phase which is attributed to an increased β volume fraction within the alloys. Furthermore, this has an influence on the mechanical properties. Thus, the Ti-25Ta alloy exhibits the highest hardness, HV353 ± 28, and the lowest value of the elastic modulus (62 GPa), which is very close to that of the bone. The experimental data confirm a very good behavior in the studied Ti-xTa alloys in simulated fever conditions and the apatite coating formed on the surface further improves the excellent biocompatibility of these alloys.

## Figures and Tables

**Table 1:** Dimensions of one set of samples for tensile tests.

Alloy	L_o_ [mm]	W_o_ [mm]	T_o_ [mm]	S_o_ [mm^2^]
Ti5Ta	3.21	1.52	0.34	0.51
Ti15Ta	2.31	1.35	0.26	0.35
Ti25Ta	2.76	1.61	0.25	0.41

**Table 2:** Hardness measurements on Ti-xTa samples.

Sample	Without Treatment	With NaOH Treatment
Ti-5%Ta	155 ± 24	212 ± 17
Ti-15%Ta	167 ± 03	224 ± 62
Ti-25%Ta	219 ± 39	353 ± 28

## References

[1] Acar M.T., Kovacı H., Çelik A. (2022). Investigation of Corrosion and Tribocorrosion Behavior of Boron Doped and Graphene Oxide Doped TiO_2_ Nanotubes Produced on Cp-Ti. Mater. Today Commun..

[2] Jamari J., Ammarullah M.I., Santoso G., Sugiharto S., Supriyono T., van der Heide E. (2022). In Silico Contact Pressure of Metal-on-Metal Total Hip Implant with Different Materials Subjected to Gait Loading. Metals.

[3] Nicholson J.W. (2020). Titanium Alloys for Dental Implants: A Review. Prosthesis.

[4] Dias Corpa Tardelli J., Bolfarini C., Cândido dos Reis A. (2020). Comparative Analysis of Corrosion Resistance between Beta Titanium and Ti-6Al-4V Alloys: A Systematic Review. J. Trace Elem. Med. Biol..

[5] Niinomi M., Liu Y., Nakai M., Liu H., Li H. (2016). Biomedical Titanium Alloys with Young’s Moduli Close to That of Cortical Bone. Regen. Biomater..

[6] Chen Q., Thouas G.A. (2015). Metallic Implant Biomaterials. Mater. Sci. Eng. R Rep..

[7] Baltatu M.S., Vizureanu P., Sandu A.V., Florido-Suarez N., Saceleanu M.V., Mirza-Rosca J.C. (2021). New Titanium Alloys, Promising Materials for Medical Devices. Materials.

[8] Gordin D.M., Delvat E., Chelariu R., Ungureanu G., Besse M., Laillé D., Gloriant T. (2008). Characterization of Ti-Ta Alloys Synthesized by Cold Crucible Levitation Melting. Adv. Eng. Mater..

[9] De Souza K.A., Robin A. (2003). Preparation and Characterization of Ti-Ta Alloys for Application in Corrosive Media. Mater. Lett..

[10] Dhinasekaran D., Kaliaraj G.S., Jagannathan M., Rajendran A.R., Prakasarao A., Ganesan S., Subramanian B. (2021). Pulsed Laser Deposition of Nanostructured Bioactive Glass and Hydroxyapatite Coatings: Microstructural and Electrochemical Characterization. Mater. Sci. Eng. C.

[11] Lin M.H., Chen Y.C., Liao C.C., Lin L.W., Chen C.F., Wang K.K., Chen S.T., Hsueh Y.H., Wu C.H., Ou S.F. (2022). Improvement in Bioactivity and Corrosion Resistance of Ti by Hydroxyapatite Deposition Using Ultrasonic Mechanical Coating and Armoring. Ceram. Int..

[12] Ganvir A., Nagar S., Markocsan N., Balani K. (2021). Deposition of Hydroxyapatite Coatings by Axial Plasma Spraying: Influence of Feedstock Characteristics on Coating Microstructure, Phase Content and Mechanical Properties. J. Eur. Ceram. Soc..

[13] Henao J., Sotelo-Mazon O., Giraldo-Betancur A.L., Hincapie-Bedoya J., Espinosa-Arbelaez D.G., Poblano-Salas C., Cuevas-Arteaga C., Corona-Castuera J., Martinez-Gomez L. (2020). Study of HVOF-Sprayed Hydroxyapatite/Titania Graded Coatings under in-Vitro Conditions. J. Mater. Res. Technol..

[14] Oladijo S.S., Akinlabi E.T., Jen T.C., Mwema F.M., Oladijo O.P. (2022). Effect of Power and Deposition Time on Sputtered Hydroxyapatite Thin Film Coatings on Stainless Steel 304. Mater. Today Proc..

[15] Kazemi M., Ahangarani S., Esmailian M., Shanaghi A. (2022). Investigating the Corrosion Performance of Ti-6Al-4V Biomaterial Alloy with Hydroxyapatite Coating by Artificial Neural Network. Mater. Sci. Eng. B Solid-State Mater. Adv. Technol..

[16] Kim C., Kendall M.R., Miller M.A., Long C.L., Larson P.R., Humphrey M.B., Madden A.S., Tas A.C. (2013). Comparison of Titanium Soaked in 5 M NaOH or 5 M KOH Solutions. Mater. Sci. Eng. C.

[17] Jalota S., Bhaduri S., Bhaduri S.B., Tas A.C. (2007). A Protocol to Develop Crack-Free Biomimetic Coatings on Ti6Al4V Substrates. J. Mater. Res..

[18] Wang C.X., Wang M., Zhou X. (2003). Nucleation and Growth of Apatite on Chemically Treated Titanium Alloy: An Electrochemical Impedance Spectroscopy Study. Biomaterials.

[19] He D.H., Wang P., Liu P., Liu X.K., Ma F.C., Zhao J. (2016). HA Coating Fabricated by Electrochemical Deposition on Modified Ti6Al4V Alloy. Surf. Coat. Technol..

[20] De Oliveira M.G., Radi P.A., Pereira Reis D.A., Dos Reis A.G. (2021). Titanium Bioactive Surface Formation via Alkali and Heat Treatments for Rapid Osseointegration. Mater. Res..

[21] Welsch G., Boyer R., Collings E.W. (1994). Materials Properties Handbook: Titanium Alloys.

[22] Yumak N., Aslantas K. (2020). A Review on Heat Treatment Efficiency in Metastable b Titanium Alloys: The Role of Treatment Process and Parameters. J. Mater. Res. Technol..

[23] Hulka I., Florido-Suarez N.R., Mirza-Rosca J.C., Saceleanu A. (2022). Ti–Ta Dental Alloys and a Way to Improve Gingival Aesthethic in Contact with the Implant. Mater. Chem. Phys..

[24] Zhou Y.L., Niinomi M., Akahori T. (2004). Effects of Ta Content on Young’s Modulus and Tensile Properties of Binary Ti-Ta Alloys for Biomedical Applications. Mater. Sci. Eng. A.

[25] Suttner S., Merklein M. (2017). A New Approach for the Determination of the Linear Elastic Modulus from Uniaxial Tensile Tests of Sheet Metals. J. Mater. Process. Technol..

[26] Zhou Y.L., Niinomi M. (2009). Ti-25Ta Alloy with the Best Mechanical Compatibility in Ti-Ta Alloys for Biomedical Applications. Mater. Sci. Eng. C.

[27] Lord J.D., Morrell R.M. (2010). Elastic Modulus Measurement—Obtaining Reliable Data from the Tensile Test. Metrologia.

[28] Mareci D., Chelariu R., Gordin D.M., Ungureanu G., Gloriant T. (2009). Comparative Corrosion Study of Ti-Ta Alloys for Dental Applications. Acta Biomater..

[29] Aniołek K., Kupka M., Barylski A., Mieszczak Ł. (2016). Characteristic of Oxide Layers Obtained on Titanium in the Process of Thermal Oxidation. Arch. Metall. Mater..

[30] Vayenas C.G., White R.E., Gamboa-Aldeco M.E. (2007). Modern Aspects of Electrochemistry.

[31] Socorro-Perdomo P.P., Florido-Suárez N.R., Mirza-Rosca J.C., Saceleanu M.V. (2022). EIS Characterization of Ti Alloys in Relation to Alloying Additions of Ta. Materials.

[32] Kizuki T., Takadama H., Matsushita T., Nakamura T., Kokubo T. (2010). Preparation of Bioactive Ti Metal Surface Enriched with Calcium Ions by Chemical Treatment. Acta Biomater..

[33] Kim H.M., Kaneko H., Kokubo T., Miyazaki T., Nakamura T. (2003). Mechanism of Apatite Formation on Bioactive Tantalum Metal in Simulated Body Fluid. Key Eng. Mater..

